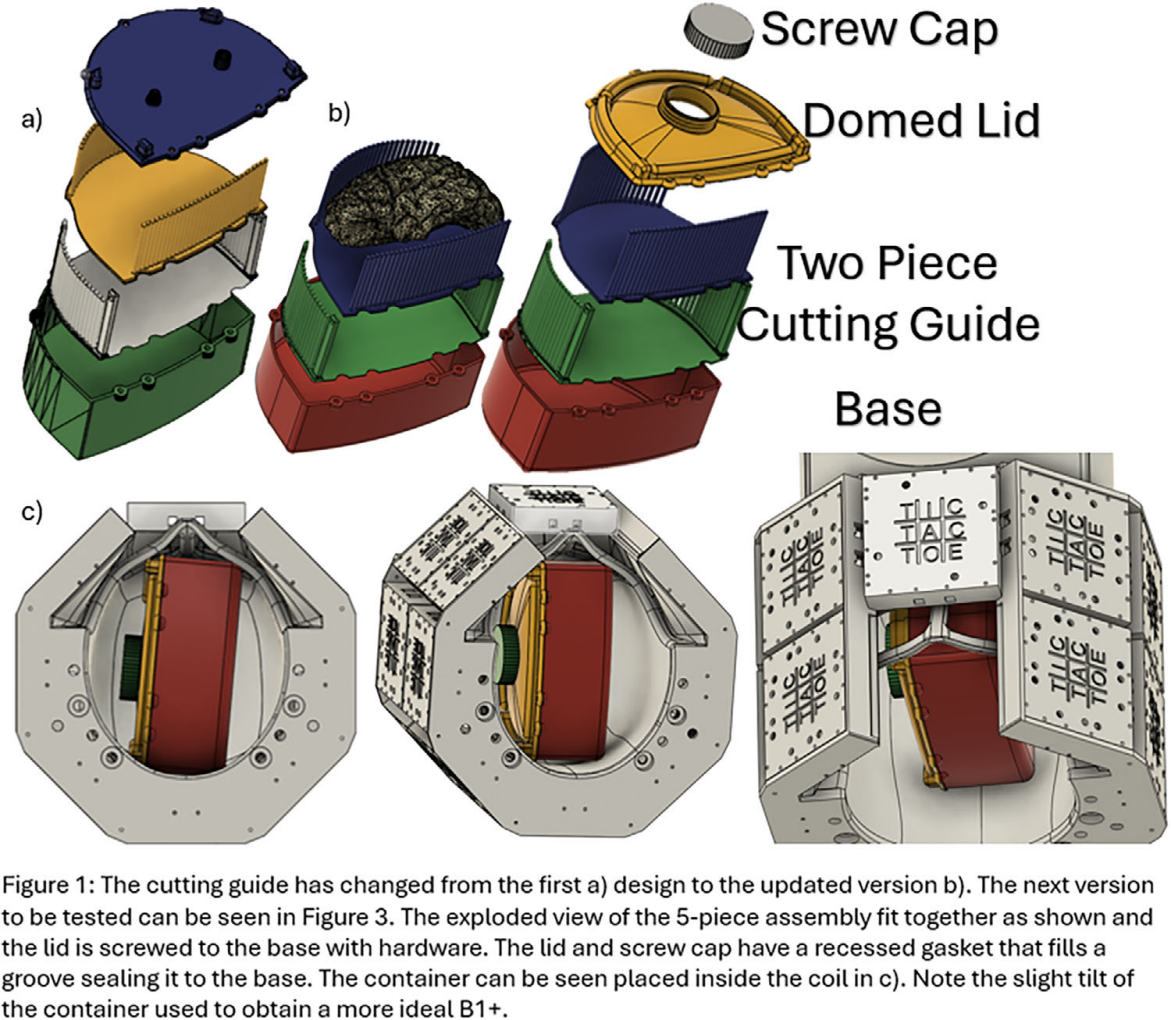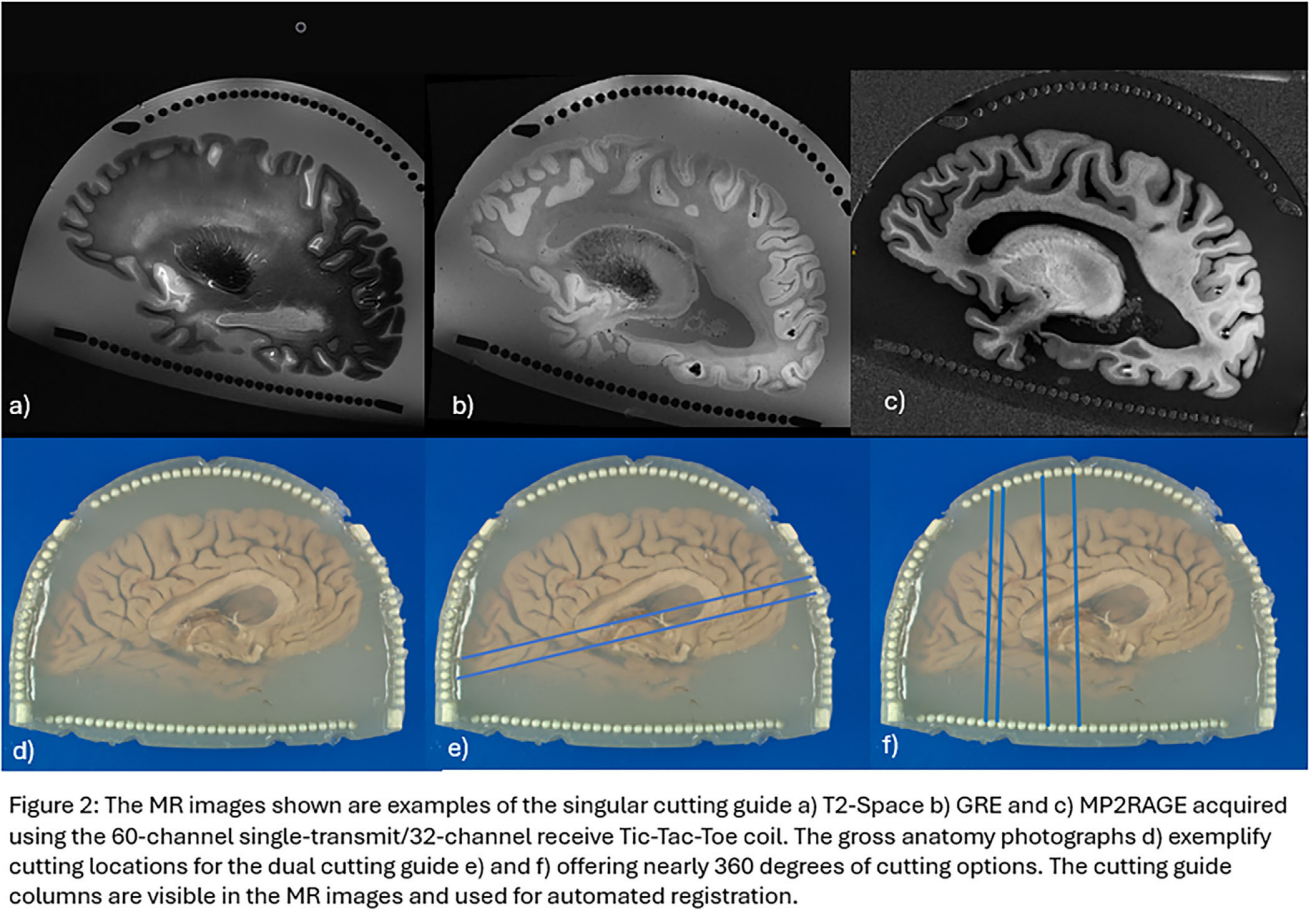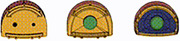# Advancements in High‐Resolution MRI Postmortem brain imaging: Histological examination on cells exclusively exhibiting White Matter Hyperintensities made possible using a novel, 3D printed, reusable, imaging container and cutting guide

**DOI:** 10.1002/alz.094152

**Published:** 2025-01-09

**Authors:** Jacob Berardinelli, Jinghang Li, Nadim Farhat, Jr‐Jiun Liou, Andrea Sajewski, Tales Santini, Minjie Wu, Joseph Mettenburg, Howard J Aizenstein, Milos D Ikonomovic, Julia Kofler, Tamer S Ibrahim

**Affiliations:** ^1^ University of Pittsburgh, Pittsburgh, PA USA; ^2^ UPMC, Pittsburgh, PA USA

## Abstract

**Background:**

Employing a custom 60‐channel single‐transmit/32‐channel receive Tic‐Tac‐Toe (TTT) coil in 7T MRI imaging has enhanced the resolution and contrast for identifying White Matter Hyperintensities (WMHs), which are critical markers in Alzheimer’s disease and related dementias (AD/ADRD).

**Method:**

The method involves resecting the left hemisphere of the brain, excluding the cerebellum, followed by embalming in 10% formalin. 1.5% agar and 30% sucrose by weight are heated and mixed for embedding in the container. The brain is positioned in the cutting guide and inserted into the base (Figure 1). Agar is meticulously poured ensuring minimal air bubble formation in the sulci and ventricles with additional agar pipetted as needed. The lid is then attached, more agar is added until the container is filled, and the cap is secured. The container is sealed in plastic to safeguard the coil from potential liquid damage and isolate the tissue/formalin while scanning. The imaging protocol includes a B1+ map at 3.2mm, MP2RAGE at 0.4mm, GRE at .37mm, and T2‐SPACE at .36mm all isotropic resolutions. The high‐resolution T2‐weighted SPACE is used for WMH segmentation and quantification. The MP2RAGE provides a T1‐weighted image for further data analysis registration to gross anatomy as well as in vivo scans.

**Result:**

This protocol has been applied to 120 brain specimens to date, with representative MR images in Figure 2. The techniques afford pathologists flexibility in slicing the brain into coronal or axial parallel 5 mm slabs at various angles. See Figure 3 for a visual representation of the progression of the container design and of the cutting process.

**Conclusion:**

This innovative combination of the cutting guide, container, and the custom 7T TTT coil is reusable and reliable. It accommodates various brain sizes, offers precise cutting options, ensures fast and accurate image registration to in vivo and gross anatomy, and achieves homogeneous B1 fields, culminating in superior research data. The described method is subject to ongoing refinement for enhanced efficiency, with iterative optimization of the container’s shape, size, and function through 3D printing. This work was supported by the National Institutes of Health under award numbers R01MH111265, R01AG063525, T32MH119168, and U19AG068054.